# Connectivity sustains disease transmission in environments with low potential for endemicity: modelling schistosomiasis with hydrologic and social connectivities

**DOI:** 10.1098/rsif.2008.0265

**Published:** 2008-09-09

**Authors:** David Gurarie, Edmund Y.W. Seto

**Affiliations:** 1 Department of Mathematics, Case Western Reserve University 10900 Euclid Avenue, Cleveland, OH 44122, USA; 2 School of Public Health, University of California 140 Warren Hall, Berkeley, CA 94720, USA

**Keywords:** metapopulations, connectivity, network, mathematical modelling, infectious diseases, disease control

## Abstract

Social interaction and physical interconnections between populations can influence the spread of parasites. The role that these pathways play in sustaining the transmission of parasitic diseases is unclear, although increasingly realistic metapopulation models are being used to study how diseases persist in connected environments. We use a mathematical model of schistosomiasis transmission for a distributed set of heterogeneous villages to show that the transport of parasites via social (host movement) and environmental (parasite larvae movement) pathways has consequences for parasite control, spread and persistence. We find that transmission can be sustained regionally throughout a group of connected villages even when individual village conditions appear not to support endemicity. Optimum transmission is determined by an interplay between different transport pathways, and not necessarily by those that are the most dispersive (e.g. disperse social contacts may not be optimal for transmission). We show that the traditional targeting of villages with high infection, without regard to village interconnections, may not lead to optimum control. These findings have major implications for effective disease control, which needs to go beyond considering local variations in disease intensity, to also consider the degree to which populations are interconnected.

## 1. Introduction

The spread of infectious diseases depends upon interactions between infectious agents, hosts, vectors and environmental reservoirs. The movement of infectious agents from one population to another can occur via various transport processes, including both host movement (e.g. migration) and physical transport processes. Numerous strategies, including quarantines and travel and trade restrictions, aim to control the spread of disease by restricting these interactions. Such controls are often costly, creating debate over the efficacy and appropriateness of various strategies (Barbera *et al*. 2001; Woolhouse & Donaldson 2001; Leuck *et al*. 2004; World Health Organization Department of Communicable Disease Surveillance and Response 2004; Ooi *et al*. 2005). Recent studies have shown metapopulation modelling to be a powerful tool in understanding the persistence and patterning of diseases, and exploring the efficacy of disease control strategies in realistic social groups such as families, villages and cities (Grenfell & Harwood 1997; Hagenaars *et al*. 2004). Here, we consider the persistence of the parasitic disease schistosomiasis as a particularly well-suited example for metapopulation modelling owing to the parasites' ability to persist in many environments despite on-going control programmes and the complexity of its transmission, in which multiple opportunities for connectivity exist (Liang *et al*. 2007).

Schistosomiasis, or bilharzia, is a waterborne parasitic disease that affects approximately 200 million people in 74 countries, primarily in sub-Saharan Africa, but also in some areas of Asia and Latin America (Chitsulo *et al*. 2000). There are five species of the *Schistosoma* parasite that cause disease in humans: *Schistosoma mansoni*, *S. intercalatum*, *S. haematobium*, *S. japonicum* and *S. mekongi*. An estimated 20 million people have severe symptoms from the disease. Of the tropical diseases, only malaria accounts for a greater global burden than schistosomiasis (Savioli *et al*. 2002). The life cycle of the parasite is described in figure 1.

For schistosomiasis there is increasing evidence that connectivity via physical processes allows for disease spread and persistence. An example of one such process is the introduction of irrigation networks, through which schistosomiasis transmission can increase and spread into previously non-endemic areas. Globally, an estimated 63 million people at risk for schistosomiasis live in irrigated environments (Steinmann *et al*. 2006). On average, based on studies from around the world, these environments increase the relative risk of urinary and intestinal schistosomiasis by 1.1 and 4.7, respectively, compared with non-irrigated environments (Steinmann *et al*. 2006). In the case of the Diama dam in Senegal, the development of irrigation channels following the construction of the dam resulted in increased transmission of *S. haematobium* and the introduction of *S. mansoni* (Talla *et al*. 1990; Picquet *et al*. 1996). Conversely, in Madagascar the destruction of the Dabara dam and its associated irrigation network resulted in reductions of *S. mansoni* transmission in the absence of any systematic chemotherapy treatment (Ravaoalimalala *et al*. 1995).

Parasite transport via social connections may also provide a pathway for schistosomiasis transmission. Visitors to endemic areas may become infected. For instance, there have been reports that visitors to endemic areas in Malawi have acquired *S. haematobium* infection (Cetron *et al*. 1996). In China, soldiers sent to endemic areas for flood relief have become heavily infected (Ross *et al*. 2001). Once infected, these individuals may introduce the parasite to new areas (if suitable snail hosts exist). Numerous studies have documented the levels of infection in migrant populations. For instance, *S. mansoni* infection in migrants from Mozambique entering KwaZulu-Natal in northeastern South Africa was found to be seven times higher than that in local residents (Appleton *et al*. 1996). In the Gezira area of Sudan, more than half of migrant labourers were found to be infected with *S. mansoni* (Bella *et al*. 1980). While these studies illustrate that infected individuals carry their parasites with them when they travel, whether this can initiate or alter local transmission depends upon numerous conditions (e.g. presence of an appropriate intermediate host, exposure, poor sanitary conditions). In this regard, some interesting evidence comes from studies in Brazil that have documented the relationship between migration and the presence of urban schistosomiasis—local transmission that has been found in areas of poor sanitation in an urban environment (Ximenes *et al*. 2000). Migrants to the urban municipio that came from endemic areas had the same level of infection risk as natives, while the risk for those coming from non-endemic areas increased with time spent in the municipio. In China, there is evidence that *S. japonicum* transmission has been altered in certain areas impacted by mobile labour and population relocations due to water resource projects (Li *et al*. 2007).

Mathematical models have been useful in understanding the transmission characteristics of parasitic diseases. However, such models often assume that populations are homogeneous (Halloran 2001), and hence do not account for parasite transport via social or physical connections. An alternative modelling approach that may be better suited to studying the spread of diseases is to treat disease transmission as a spatially realistic metapopulation problem, in which infectious agents persist within a network of connected environments (Hanski 2001). The two aforementioned forms of parasite transport (social and physical) can be accommodated with such models. For schistosomiasis, hydrological transport is an important physical process with directionality, i.e. it transports larval forms of the schistosome parasite from upstream to downstream villages. By contrast, human social behaviour allows for bidirectional parasite transport, i.e. hosts can become infected and contribute parasitic eggs both within and outside of their own village.

Different patterns of connectivity in distributed environments require different approaches. A few studies (e.g. Barbour 1978; Woolhouse 1991; Gurarie & King 2005) have discussed distributed networks of human–snail contact sites, and the effect of such heterogeneity. Woolhouse, in particular, has called for improved modelling of these concepts. In this paper we focus on a different connectivity pattern that provides a fairly realistic representation of a region made up of endemic villages, whereby all human–snail–larval contacts are local, yet environmental connections link a distributed metapopulation through hydrology (as a means of larval transport) and social interactions (direct transport of infection by human hosts).

Specifically, we present a connected model of schistosomiasis transmission that accounts for parasitic transport between villages, and use this model to show that transport can impact parasite control, spread and persistence. In particular, we discuss the findings related to design of effective schistosomiasis control programmes.

## 2. Methods

We start with a brief overview of the classic Macdonald model of schistosomiasis transmission (Macdonald 1965) for a homogeneous population. The parameters of this model are presented in table 1. The basic model considers a single human population made up of *H* individuals who live in an environment with *N* intermediate snail hosts. Both quantities *H* and *N* can be viewed as total populations, or more appropriately population densities per ‘unit contact area’ of habitat. Schistosomiasis infection is quantified for this population by the mean number of schistosomes per person, *w*, and the prevalence (proportion) of infectious snails, *y*. Additionally, we use variables *c* and *m* for the cercarial and miracidial larval densities, respectively.

The rates of change in these four variables may be expressed as a system of differential equations as follows:

(2.1) $$\begin{array}{ll} \frac{\text{d}w}{\text{d}t}=a\theta c-\gamma w; & \frac{\text{d}y}{\text{d}t}=bm(1-y)-\nu y;\\ \frac{\text{d}c}{\text{d}t}=\pi_{\text{C}}Ny-\mu_{\text{C}}c; & \frac{\text{d}m}{\text{d}t}=\pi_{\text{M}}\theta^{\prime }H\frac{w}{2}-\mu_{\text{M}}m. \end{array}\}$$

Briefly, each of the above equations consists of the rates at which parasites or infected populations are gained or lost. For example, in the first equation in (2.1), the change in the mean number of schistosomes per person consists of a gain term *aθc*, and a loss term due to death of adult worms, *γw*. The former *aθc* represents the rate of burden accumulation resulting from a given cercarial density and contact (exposure) rate. Similarly, for the second equation in (2.1), the gain of snail infection is equal to the product of miracidial density and the prevalence of uninfected snails *bm*(1−*y*), while the loss is due to their natural mortality (1/*ν* is the average life expectancy of a snail). The cercarial and miracidial equations behave similarly with gain resulting from infected snail/human hosts and loss from natural mortality.

Numerous simplifying assumptions are made in this model. First, underlying human/snail populations are assumed to be constant (i.e. deaths=births). This may be acceptable for humans, yet unrealistic for snail populations given real-world fluctuations by season and year. Another significant limitation is homogeneity of human–snail contacts and associated risk factors for infection. Some models (e.g. Anderson & May 1991) account for latency in infection in the intermediate host in order to reflect better the lower levels of snail prevalence that are observed in the field due to snail mortality during this period. However, the fractions of prepatent infected snails and patent infectious snails are approximately proportional to one another, as typically a specific (small) fraction of infected snails are converted to patency (10% or less). Hence, we did not explicitly model the latency. Also note that factor *w*/2 in the miracidial production represents the (approximate) number of mated worm pairs in human hosts, assuming a balanced ratio of male to female worms (May 1977). Moreover, human worm burden is distributed unevenly (overdispersed). Hence, more refined models account for this uneven distribution by multiplying *w*/2 by a suitable *mating probability* factor 0<*ϕ*(*w*)<1 (itself a function of *w*) as well as an aggregation parameter that together describes the distribution of worms among hosts. Henceforth, we assume that the factor of two is encapsulated into *b* for simplicity.

We also comment on different contact rates *θ*, *θ*′ for exposure and contamination. Their form depends on the specifics of human–snail interactions in different communities and environments. In some locations (e.g. eastern Kenya; Gurarie & King 2005) the same contacts can lead to both exposure and contamination (so *θ*≈*θ*′). In other places (e.g. China; Spear *et al*. 2004; Seto *et al*. 2007*a*), a different pattern takes place whereby all infected hosts can contribute to contamination (through the use of human excrement for fertilizer), but their exposure may differ depending on occupational risk (*θ*≠*θ*′).

While these modifications can be important in transmission dynamics, and the persistence and endemicity of human–snail systems, here we shall confine ourselves to the basic transmission model (2.1), as our main focus lies in unravelling the role of connectivity. Having understood the effect of connectivity for simple transmission patterns, we intend to expand our study to more realistic systems, and apply it to field data on connectivity and epidemiology in future work.

Coming back to system (2.1), the parasite larval stages (represented by densities *c* and *m*) have relatively short lifespans compared with those of worms and snails. So typically, the dynamic equations for *c* and *m* are replaced with their quasi-equilibrated values, $c^{*}=(\pi_{\text{C}}/\mu_{\text{C}})Ny$ and $m^{*}=(\pi_{\text{M}}/\mu_{\text{M}})\theta^{\prime }Hw$. Then the original system (2.1) is reduced to a two-dimensional form for variables *w*, *y*

(2.2) $$\frac{\text{d}w}{\text{d}t}=Ay-\gamma w,\;\;\frac{\text{d}y}{\text{d}t}=Bw(1-y)-\nu y,$$

where coefficients *A* and *B* represent ‘snail–human’ and ‘human–snail’ transmission, respectively:

(2.3) $$A=a\frac{\pi_{\text{C}}}{\mu_{\text{C}}}\theta N;\;\;B=b\frac{\pi_{\text{M}}}{\mu_{\text{M}}}\theta^{\prime }H.$$

Systems (2.1) or (2.2) define the basic reproduction number *R*_0_, a dimensionless parameter

(2.4) $$R_{0}=\frac{AB}{\gamma \nu },$$

which establishes a criterion for the dynamic behaviour of the disease transmission system: a *stable endemic equilibrium* if *R*_0_>1, and *elimination* (stable ‘zero’ equilibrium *w*=*y*=0) if *R*_0_<1. Indeed, equilibrium system (2.2) gives

(2.5) $$R_{0}y=\frac{y}{1-y};\;\;w=\frac{A}{\gamma }y.$$

As *R*_0_ is explicitly defined by transmission and mortality rates, as well as human and vector populations, it allows one to qualitatively examine the effects of various control interventions.

With the basic model and the concept of *R*_0_ described, we now extend the Macdonald model to a distributed metapopulation system made of separate, but connected, villages, each one with its own snail habitat. We consider two types of connectivity: hydrology (a directional transport process that carries free-living cercarial and miracidial larval stages of the parasite) and social contact (a diffusive transport process that spreads infection in many directions via the two following processes: human hosts both becoming infected and contaminating their own and other villages).

### 2.1 Hydrological and social connectivities

Hydrology is assumed to be a system of volumes (reservoirs) {*V*_*i*_} connected via unidirectional channels that transport the flow of parasite larvae (cercariae and miracidia) from upstream to downstream villages at certain throughput rates {*r*_*ij*_} (per unit volume). We assume volumes, discharge/loss rates, sources and sinks to be stationary in time, although we shall study the effect of varying levels of mean transport (‘fast’ versus ‘slow’). Such a simplified model still retains some essential features of complex hydrologic processes, and allows qualitative analysis of hydrologic factors on the spread of infection.

We are primarily interested in the hydrologic transport of larval densities, whereby a fraction of *c* and *m* at site *j* is transported to downstream site *i*. Such transport operates roughly at similar timescales as the lifespan of larvae (hours). Thus, only a fraction of larvae will survive to the final destination. This survival fraction should depend on the travel time, which is (approximately) proportional to the distance between two sites *d*_*ij*_ and inversely proportional to mean velocity of the channel flow. The distance and velocity in turn may depend on specifics of topography and environment of a given area. Here, we assume approximate geographical distances between up/down stream locales (figure 2), and make the survival fraction equal to ${\text{e}}^{-\beta d_{ij}}$ with suitable coefficients *β*_C_ and *β*_M_, denoting different *β* for cercariae and miracidia, respectively (the latter having shorter lifespan). Survival exponents *β*_C/M_ are independent of the throughput rate *r*. Thus, two essential parameters in our hydrologic scheme are the ‘survival exponent’ *β* and relative throughput rates {*r*_*ij*_}.

The above scheme gives a somewhat simplified view of transport and survival, which can be modified in many ways to accommodate more realistic topography, environment and biology. We also note that hydrology may transport snails along with larval stages for some endemic areas, and such transport can be included in our scheme. But in what follows, we shall primarily focus on larval transport because the adult snail host in China (where much of our epidemiologic research is conducted) dies when submerged in water, and thus may not be subject to hydrologic transport to the same degree as other snail species.

Field data from cercarial release experiments in China provide some insight into plausible values for the *β* transport coefficient (Lowe *et al*. 2005). Using cages of mice placed in an irrigation ditch at several distances downstream of a single cercarial release point, the decay in infective cercariae with distance was reasonably approximated by an exponential decay with an approximate half-life distance of 50 m (*β*=0.014 m^−1^). Since miracidia have a shorter lifespan, it may be reasonable to assume that they decay at a faster rate.

Social contacts are assumed to follow a gravity model—one of the most widely used spatial models of social behaviour (Haynes & Fotheringham 1984) in which local populations serve as attractive masses, and contact rates decrease with distance. For social contacts we assume hosts from site *j* visit site *i* at a rate *θ*_*ij*_. At each contact a human host can become infected, and/or contaminate the site in proportion to his/her contact rate. As discussed before for the basic Macdonald model, it is possible to differentiate between contact rates for the infection and contamination processes (i.e. *θ*_*ij*_ versus $\theta_{ij}^{\prime }$). To determine the dispersion of contacts among villages, we assume that the population size at each node *H*_*i*_ serves as an *attraction* (to the village), which decreases exponentially with distance (i.e. distance between the two sites creates a geographical *hurdle*) according to the function Const×e^−*αd*^ parametrized by the factor *α*. Hence, the total (site specific) contact rate at the *j*-th village *θ*_*j*_ gets redistributed among all other sites in proportion to $\left\{H_{i}{\text{e}}^{-\alpha d_{ij}}:i=1,2,\ldots \right\}$. We call the corresponding relative contact fractions $\omega_{ij}=\theta_{ij}/\theta_{j}\;(\sum_{i}\omega_{ij}=1)$ and $\left\{\theta_{ij}=\omega_{ij}\theta_{j}\right\}$. We note that the social contact dispersion coefficient *α* (as *β* for hydrology) is inversely related to the ‘strength’ of connectivity, with high values representing relatively dispersed social behaviour, and small values localized social behaviour.

Again, data from China provide some insight into the applicability of the social contact model. While not a direct model of social interaction, Benziger (1996) modelled relatively large-scale urban–rural interactions as they pertained to rural productivity. A gravity model estimated rural productivity as a function of an attractive mass that promotes growth (industrial output in nearby cities) and a geographical hurdle (distance to the nearby cities). At an even larger spatial scale, gravity models have also been used to study interprovincial migration using employment opportunity as an attractor and distance as a hurdle (Fang & Dewen 2003). At the village level, data from a study of villagers wearing global positioning system receivers documented frequent travel outside of one's own village, typically along road networks, to population centres (e.g. township centres and rural markets; Seto *et al*. 2007*b*). We reinterpreted the GPS data to enumerate the person-trips made, and the distances to different locations. From this, we estimated that the plausible social contact exponent for our schistosomiasis endemic area in China to be approximately *α*=0.0005 m^−1^.

### 2.2 Connected model of schistosomiasis transmission

With the connectivities characterized, we now describe the human–snail–larvae transmission model—an extended metapopulation version of system (2.1) in a connected environment. The variables and parameters of table 1 are now made site specific, e.g. human–snail–larval densities: $H_{i};N_{i};c_{i};m_{i}$, as well as mean worm burdens and snail infection prevalences: *w*_*i*_; *y*_*i*_ for a given site, *i*. We augment these with additional transport-connectivity parameters that are specific to the metapopulation system (table 2). All environmental and demographic variables, including human and snail populations, are assumed as stationary in time.

For a connected set of *n* sites (villages), the four-dimensional Macdonald model (2.1) becomes a global 4*n*-dimensional system:

(2.6) $$\frac{\text{d}w_{i}}{\text{d}t}=a\theta_{i}\sum_{j}c_{j}\omega_{ji}-\gamma w_{i};\frac{\text{d}y_{i}}{\text{d}t}=bm_{i}(1-y_{i})-\nu y_{i};\frac{\text{d}c_{i}}{\text{d}t}=\overset{\pi_{\text{C}}N_{i}y_{i}}{\underset{\text{Local}\;\text{production}}{︸}}+\overset{\sum_{j<i}s_{ij}^{\text{C}}r_{ij}\frac{V_{j}}{V_{i}}c_{j}}{\underset{\text{Influx}}{︸}}-\overset{(\varepsilon r_{i}+\mu_{\text{C}})c_{i}}{\underset{\text{Outflux}+\text{mortality}}{︸}};\frac{\text{d}m_{i}}{\text{d}t}=\pi_{\text{M}}\sum_{j}\omega_{ij}\theta_{j}^{\prime }H_{j}w_{j}+\sum_{j<i}s_{ij}^{\text{M}}r_{ij}\frac{V_{j}}{V_{i}}m_{j}-(\varepsilon r_{i}+\mu_{\text{M}})m_{i}.$$

This system is derived from balance relations for all relevant quantities (snail, worm, larval densities) that account for their sources (growth, production) and sinks (losses). We shall comment on a peculiar form of the ‘discharge (loss) fraction’ terms *ϵr*_*i*_ in the *c* and *m* equations. For conservative species in a typical mixing-transport network made of interconnected nodes, the local balance requires a discharge rate, $r_{i}=\sum_{i≺j}r_{ji}$, be equal to the cumulative outflux from site *i* to all downstream locations *j* (here i≺j indicates *j* downstream of *i*). So one would expect *ϵ*=1 in the *c*, *m* equations of (2.6). However, a number of biological factors may reduce the apparent larval outflow at each site, and thus make *ϵ* smaller. Among them are ‘resource limitations’ for larval survival and development and ‘density-dependent effects’ (e.g. clumping of pathogens and subsequent clinging to within-channel vegetation, common for waterborne species). Without providing a full account of such processes, we hypothesize their net effect to be a reduced larval ‘outflow discharge loss’, a small fraction *ϵ* of *r*_*i*_. To put it succinctly, the out-flowing larval density (at rate *r*_*i*_) is replenished through local production to maintain the same level as it would attain with all outflow channels shut down, provided this level is close to the natural carrying capacity of reservoir.

The above model has some similarities with earlier versions of the distributed human–snail–schistosome environment (e.g. Woolhouse 1991; Gurarie & King 2005). Woolhouse's formulation is based on earlier work by Barbour (1978), which considers separated human and snail sites. Their focus is on understanding individual-level differences in spatial distribution of water contact. In Gurarie & King (2005), we use ‘geographical hurdle’ to parametrize human–snail contact patterns in a distributed environment. The main difference and novelty of the current model is a combination of both human contact/contamination patterns and parasite transport via hydrologic connectivity.

Our goal is to explore the behaviour of system (2.6), its equilibria and the conditions required for the persistence of transmission. We then study the effect of varying levels of hydrological and social connectivity. Finally, we consider how the model performs under typical disease control strategies. All the computational procedures below are implemented in Wolfram Mathematica (version 5 and 6).

## 3. Results

### 3.1 Equilibrium and criteria for disease persistence

Just as the Macdonald system (2.1) above was reduced to a simpler two-dimensional form (2.2), system (2.6) can be reduced to a ‘worm-burden+infected snail prevalence’ form—an extended version of (2.2). Again, we use the relatively short lifespan of the larval stages, which leads to a quasi-equilibration of the *c* and *m* equations. Using an abbreviated notation, we recast system (2.6) into a matrix-vector form for variables: $W=(w_{i});\;Y=(y_{i});\;C=(c_{i});\;M=(m_{i})$, as

(3.1) $$\frac{\text{d}W}{\text{d}t}=a\hat{\Theta }\cdot \Omega^{\text{T}}\cdot C-\gamma W;\frac{\text{d}Y}{\text{d}t}=bM(1-Y)-\nu Y;\frac{\text{d}C}{\text{d}t}=\pi_{\text{C}}\hat{N}\cdot Y-T_{\text{C}}\cdot C;\frac{\text{d}M}{\text{d}t}=\pi_{\text{M}}\Omega \cdot ({\hat{\Theta }}^{\prime }\cdot \hat{H})\cdot W-T_{\text{M}}\cdot M.\}$$

Here, we use the following notation:

- ‘hat-label’=the diagonal matrix of an array, e.g. $\hat{N}=\text{diag}(N_{1},\ldots ,N_{n})$, or $\hat{\Theta }=(\theta_{1},\ldots ,\theta_{n});{\hat{\Theta }}^{\prime }=(\theta_{1}^{\prime },\ldots ,\theta_{n}^{\prime })$, etc.,
- 1−*Y* abbreviates vector $\left(\ldots ,1-y_{i},\ldots \right)$,
- ‘simple product’ as *M*(1−*Y*) designates coordinate-wise multiplication of vectors, $\left\{\ldots ,m_{i}(1-y_{i}),\ldots \right\}$,
- ‘dot’ designates the usual matrix/vector product, and
- superscript T notation designates the transpose of the matrix.

Other parameters of (3.1) include lower triangular transport matrices (with rows *i* and columns *j*, and asterisks replaced by the appropriate *i*−*j* term to complete the lower triangle values):

(3.2) $$T_{\text{C}}=\left[\begin{array}{cccc} \left(\epsilon r_{1}+\mu_{\text{C}}\right) & 0 & \cdots & 0\\ {}* & \left(\epsilon r_{2}+\mu_{\text{C}}\right) & 0 & \cdots \\ {}* & -s_{ij}^{\text{C}}r_{ij}\frac{V_{j}}{V_{i}} & \ddots & 0\\ {}* & * & * & \left(\epsilon r_{n}+\mu_{\text{C}}\right) \end{array}\right],$$

for cercariae, and similarly patterned *T*_M_ for miracidia and rescaled social contact matrix *Ω*=[*ω*_*ij*_]. The ‘lower triangular’ form of the transport matrices reflects the ordering of sites according to elevation, starting with the highest one, so that for any connection where *j* lies downstream of *i* (denoted as i≺j), we have *i*<*j*. Thus, a hypothetical environment of figure 2 has the following connectivity pattern[Table tbl4]:

The quasi-equilibrium larvae solutions of system (2.6) are given by

$$C=\pi_{\text{C}}T_{C}^{-1}\cdot \hat{N}\cdot Y;\;\;M=\pi_{\text{M}}T_{\text{M}}^{-1}\cdot \Omega \cdot \hat{\Theta^{\prime }}\cdot \hat{H}\cdot W,$$

that include the infected snail prevalence vector *Y* and mean worm burden distribution *W* in the equations. Substitution of these quasi-equilibrium solutions for *C* and *M* into the *W* and *Y* equations yields the reduced Macdonald-type system:

(3.3) $$\frac{\text{d}W}{\text{d}t}=a\pi_{\text{C}}\hat{\Theta }\cdot (\Omega^{\text{T}}\cdot T_{\text{C}}^{-1})\cdot \hat{N}\cdot Y-\gamma W;\;\;\frac{\text{d}Y}{\text{d}t}=b\pi_{\text{M}}\left(T_{\text{M}}^{-1}\cdot \Omega \cdot {\hat{\Theta }}^{\prime }\cdot \hat{H}\right)\cdot (1-Y)W-\nu Y,\}$$

written in terms of inverse transport matrices $T_{\text{C}}^{-1};T_{\text{M}}^{-1}$, contact matrix *Ω* (along with its transpose *Ω*^T^) and other environmental/behavioural parameters. To explore their relative contribution to the infection potential of our environment, we recast system (3.3) into the following form:

(3.4) $$\frac{\text{d}W}{\text{d}t}=\frac{\gamma }{\nu }\left[\frac{\hat{A}}{\gamma }(R_{\text{SH}}\cdot Y)-W\right];\frac{\text{d}Y}{\text{d}t}=\left(R_{\text{HS}}\cdot \frac{\hat{B}}{\nu }\cdot W\right)(1-Y)-Y.\}$$

Here diagonal matrices $\hat{A};\hat{B}$ have entries

(3.5) $$A_{i}=\frac{a_{i}\pi_{\text{C}}\theta_{i}N_{i}}{\epsilon r_{i}+\mu_{\text{C}}};\;\;B_{i}=\frac{b_{i}\pi_{\text{M}}\theta_{i}^{\prime }H_{i}}{\epsilon r_{i}+\mu_{\text{M}}},$$

that represent local transmission factors, with the corresponding local BRN for each village given by

(3.6) $$\rho_{i}=\frac{A_{i}B_{i}}{\gamma \nu }.$$

Matrices

(3.7) $$R_{\text{SH}}={\hat{N}}_{}^{-1}\cdot \left(\Omega^{\text{T}}\cdot T_{\text{C}}^{-1}\right)\cdot \hat{N}\;\text{and}\;R_{\text{HS}}=\left(T_{\text{M}}^{-1}\cdot \Omega \right)$$

represent the contribution of social and hydrological connectivities for snail–human and human–snail transmissions, respectively. Such a formulation is notable in that it clearly separates factors related to ‘local transmission’ {*ρ*_*i*_} from those related to ‘global connectivity’ (*R*_HS_ and *R*_SH_).

Distributed systems such as (3.2)–(3.4) give rise to a matrix generalization of the fundamental BRN concept (2.4)—the so called *basic reproduction matrix* (BRM), introduced in Gurarie & King (2005). Here, it takes the form

(3.8) $$R=R_{\text{HS}}\cdot \hat{\rho }\cdot R_{\text{SH}},$$

where diagonal matrix $\hat{\rho }=\text{Diag}[\rho_{i}]$ is made up of the local BRN for each village, $\rho_{i}=A_{i}B_{i}/\gamma \nu$. Matrix *R* appears in a further reduction of the equilibrium system (3.3) for the infected snail prevalences. The latter turn out to obey the nonlinear algebraic system

(3.9) $$R\cdot Y=\frac{Y}{1-Y},$$

a ‘distributed’ version of the simple Macdonald relation (2.5). As above, ‘vector division’ *Y*/(1−*Y*) designates a coordinate-wise operation $\{y_{i}/(1-y_{i})\}.$

If we remove both types of connectivity, the transmission factors *R*_HS_ and *R*_SH_ become identity matrices. Hence, *R* turns into a diagonal matrix with just the local BRNs (for individual villages) along the main diagonal. Therefore, in a disconnected environment, endemic equilibria can only be sustained locally at sites with local BRN *ρ*_*i*_>1. In essence this brings us back to the Macdonald *R*_0_ criterion for a single-environment unconnected model.

Matrix *R* plays a role similar to *R*_0_ of equation (2.4) for connected environments. Indeed, it gives a condition for sustainable transmission in terms of its largest eigenvalue (e.g. Gurarie & King 2005),

(3.10) $$\lambda_{1}(R)>1.$$

This is a matrix analogue to *R*_0_>1. Under such a condition, matrix *R* yields a positive (endemic) stable equilibrium Y^*^ for system (3.9). The corresponding equilibrium worm burden distribution is obtained from Y^*^ via the snail–human transmission matrix $W^{*}=R_{\text{SH}}\cdot Y^{*}$.

One can think of *λ*_1_(*R*) as the *transmission potential* for a connected environment, which encapsulates the essential transmission characteristics (both local and global connectivity), albeit in a highly nonlinear implicit way, as opposed to explicit formula (2.4). 

### 3.2 Simple 2-site examples of sustained transmission via connectivity

Here, we illustrate the role of connectivity in sustaining transmission by analysing the simplest 2-village example.

We first examine the case of hydrologic connectivity without social contacts. Given two hydrologically connected sites $\begin{array}{c} 1 \end{array}\overset{C}{\underset{M}{⟶}}\begin{array}{c} 2 \end{array}$ (up/down stream), we compute the BRM of the system,

$$R=\left[\begin{array}{cc} \rho_{1} & 0\\ r_{21} & \rho_{2} \end{array}\right].$$

Its diagonal is made of local BRNs $\rho_{i}=A_{i}B_{i}/\gamma \nu$. The off-diagonal entry

(3.11) $$r_{21}=\rho_{21}\psi_{\text{M}}+\rho_{12}\psi_{\text{C}}$$

combines products of ‘BRN-like’ coefficients $\rho_{ij}=A_{i}B_{j}/\gamma \nu$, times cercarial and miracidial factors

$$\psi_{\text{C}}=\frac{N_{1}}{N_{2}}\frac{s^{\text{C}}r}{r+\mu_{\text{M}}};\;\;\psi_{\text{M}}=\frac{H_{1}\theta_{1}}{H_{2}\theta_{2}}\frac{s^{\text{M}}r}{r+\mu_{\text{C}}}.$$

The latter depend on the channel throughput *r*, local human/snail populations *H*_*i*_, *N*_*i*_, contact rates *θ*_*i*_, and larval survival probabilities *s*^C^ and *s*^M^. The resulting two-dimensional equilibrium system R·Y=Y/(1−Y) has an exact analytic solution,

(3.12) $$\begin{array}{l} y_{1}^{*}=1-1/\rho_{1}(\text{assuming}\;\rho_{1}>1)\\ y_{2}^{*}=\frac{\sqrt{{\left(1+e-\rho_{2}\right)}^{2}+4e\rho_{2}}-(1+e-\rho_{2})}{2\rho_{2}}\\ \approx \frac{e}{1+e}+\frac{e\rho_{2}}{{\left(1+e\right)}^{3}}+\cdots \end{array}\}$$

the latter $y_{2}^{*}$ being expressed through a dimensionless parameter

(3.13) $$e=r_{21}y_{1}^{*}=\left(\frac{\theta_{2}H_{2}}{\theta_{1}H_{1}}\psi_{\text{M}}+\frac{\theta_{2}N_{2}}{\theta_{1}N_{1}}\psi_{\text{C}}\right)(\rho_{1}-1).$$

Formula (3.12) demonstrates, in particular, that the downstream site has a sustained endemic level $y_{2}^{*}$, essentially independent of its local BRN *ρ*_2_. The lower bound for $y_{2}^{*}\geq (e/(1+e))$ is determined entirely by environmental factors (throughput rate, human/snail contact values at the two sites and the upstream local BRN *ρ*_1_) encapsulated in parameter *e* (3.13). Hence, even very low downstream local transmission (*ρ*_2_≈0) can sustain the endemic infection due to the influx of upstream parasites, as long as *ρ*_1_>1. This example demonstrates how hydrology can spread infection downstream, and the same principle may hold for more general connected environments with low local BRN ≪1, as we shall see below.

In a similar fashion, we can examine the effect of social connectivity in a 2-village setting. Dropping hydrologic transport from the transmission matrices *R*_SH_; *R*_HS_ (3.7), we get a 2×2 BRM $R=\Omega \cdot \hat{\rho }\cdot \Omega^{\text{T}}$, made of contact matrix *Ω* and local BRN *ρ*_*i*_,

$$\Omega =\left[\begin{array}{cc} \omega_{1} & \omega_{2}\\ \omega_{1}^{\prime } & \omega_{2}^{\prime } \end{array}\right];\;\;\omega_{i}+\omega_{i}^{\prime }=1;\;\;\hat{\rho }=\left[\begin{array}{cc} \rho_{1} & \\ & \rho_{2} \end{array}\right].$$

One can show that the largest eigenvalue

(3.14) $$\lambda_{1}(R)\leq \rho_{1}|\Omega_{1}{|}^{2}+\rho_{2}|\Omega_{2}{|}^{2};\;\;\Omega_{i}=\left(\begin{array}{l} \omega_{i}\\ \omega_{i}^{\prime } \end{array}\right)$$

can come arbitrarily close to its maximal value (the right-hand side of (3.14)) with a proper choice of vectors *Ω*_1_; *Ω*_2_. Clearly, locally unsustainable isolated sites (*ρ*_*i*_<1) can still give a sustained endemic infection in the ‘connected community’, provided that the right-hand side term ≈*λ*_1_(*R*)>1.

### 3.3 Exploring a larger connected environment

To assess the interplay between hydrologic and social connections on transmission, we explored a hypothetical distributed environment of 15 villages (figure 2). As described above, we assumed inter-village parasitic transport from both hydrologic and social processes. We decided upon this environment *a priori* of any analyses to represent an environment where the potential for transmission might be considered very low. Only one of the modelled villages (#14) had a local BRN≥1 (actually, the highest was 1 as described below). This village was located second to the lowest in elevation, and hence could only affect transmission in one downstream neighbour via hydrologic connectivity. Yet, upon analysis, we found that due to the diffusive process of social connectivity, sustained equilibrium transmission occurred in all 15 villages (figure 3).

The other parameters of our model environment are relative human, snail populations and contact rates (table 3). We also take equal volumes *V*_*i*_=1 (note that only relative volumes *V*_*i*_/*V*_*j*_ enter system (2.6)), and throughput rates *r*_*ij*_=*r*. The local BRN values $\rho_{i}=(A_{i}B_{i}/\gamma \nu )\propto H_{i}N_{i}\theta_{i}^{2}$ based on table 3 are scaled so that $\text{max}(\rho_{i})=\rho_{14}=1$. The length scale of our entire environment is equal to 1, which makes all distances *d*_*ij*_<1, and the exponents *α*, *β* scaled to 1/length for social and hydrologic connectivity. The ratio of miracidial to cercarial mortality $\mu_{\text{M}}/\mu_{\text{C}}=2$, and the dimensionless throughput rate *r*/*μ*_C_ has range [0,1].

In the real world both hydrological and social connectivity may vary with time and locale. We explored the impact of varying social and hydrological connectivities (parameters *α*, *β*, *r*) on the sustainability of transmission for the network in figure 2. We note that increased throughput rate *r* enhances inter-village connectivity, while the exponents *α*, *β* are inversely related to the strength of connectivity. Thus, large *β* corresponds to ‘slow hydrology’ (i.e. diminished hydrologic transport of larvae), and increasing *α* corresponds to higher social isolation (decreased social contact dispersion).

We found optimal combinations of the connectivity parameters that led to sustained infection (figure 4*a*). In studying this figure, recall that values of $\lambda_{1}(R)>1$ indicate sustained transmission. Generally, the potential for global transmission across the system increased with increasing hydrologic transport. Fast hydrology (*β*<6) and large throughput (*r*=1) allowed for sustained transmission over a wide range of values of the social contact dispersion *α*. However, in most cases, there existed optimum levels (neither too high nor too low) of social contact dispersion that maximized transmission globally. This suggests that regardless of the degree of hydrologic connections between villages, there is some optimum level of dispersion of social behaviour that will sustain global transmission.

Interestingly, figure 4*a* shows that as hydrologic transport decreased (throughput *r*≤0.6 and *β*=4, 5, 6, 7), transmission was not sustained for relatively dispersed social contacts (*α*<10). This suggests that as villages become hydrologically isolated, an overly mobile society may dilute transmission from the few local sites where transmission can persist in the network (i.e. in our network site 14), thereby not allowing for transmission to be sustained across the network.

Figure 4*b* provides an alternative view of these relationships. Here, the isocontours of *λ*_1_(*R*) are shown, in which the values that are greater than 1 are shaded in increasing levels of grey. Increased hydrologic throughput consistently increases the range of social connectivities that can sustain transmission. Slowing hydrology (increasing *β*) has the following two effects: decreasing the area of the grey isocontours (conditions where *λ*_1_(*R*)>1 and transmission can occur across the network) and shifting the optimal social dispersion to less dispersed contacts. Again, the optimal social conditions for global transmission for this network tend to be dispersed social behaviour when hydrology is fast (small *α*, small *β*) and localized social behaviour when hydrology is slow (larger *α*, larger *β*).

Interestingly, we note that rescaling the *β* and *α* values of 0.014 m^−1^ and 0.0005 m^−1^, respectively, which we computed from our data from China, to the unit dimensions of this hypothetical network places us in region of *β*=4, and *α*<5 (lower-right quadrant of figure 4*b*(ii)). These crude estimates of connectivity suggest that if social contacts become increasingly dispersed in the future, it may become more difficult to sustain schistosomiasis transmission.

Location within the connected network was just as important, if not more so than local BRN. This is illustrated by the distribution of equilibrium snail infection prevalence and worm burdens across the 15 villages (figure 5). In the case of slow hydrology (figure 5*a*(iii),*b*(iii)) if social contact dispersion were sufficiently constrained, the only endemic site was village #14 with BRN=1 and its downstream neighbour. For the other two levels of hydrology, the sites with the most infected snail prevalence were #5, 9, 12 and 15. For worm burdens, #2 became prominent at high social dispersal (for fast and medium transport cases), but dropped out as contacts localized. Having low local BRN, we concluded that the primary sources of infection at #2 were contacts from nearby sites #1 and 7. Other prominent sites at fast hydrology were #11 and 13, which again we would not have predicted given their relatively low local BRNs. For medium hydrology, all worm burdens were an order of magnitude lower than those for fast hydrology. Moreover, for this environment, we found that the optimal connectivities for one village may not have been the same for others. For instance, worm burden was the highest in #2 for highly dispersed contacts, while for villages #5, 9, 10 and 15, medium social dispersion was optimal.

### 3.4 Impact on disease control

Given the contradiction between local BRN and overall disease sustainability and relative amounts of transmission with distributed environments, we examined the impact of connectivity on disease control efficacy. Traditionally, and as commonsense would suggest, one might prioritize sites with highest infection. However, within a connected environment, we were able to demonstrate that such control may be inefficient. We considered for our analysis the earlier case of moderately fast hydrology *β*=5, and dispersed contacts *α*=5 (figure 3). The disease control consisted of reducing local BRN *ρ*_*i*_ (e.g. through snail control). Here the villages with the highest worm burden were #11, 12 and 13. However, as we simulated control by modifying local BRN $\left\{0<\rho_{i}<\rho_{i}^{*}:i=11,12,13\right\}$, where $\rho_{i}^{*}$ were the original BRN of figure 2, we found that $\text{min}[\lambda_{1}(\rho_{11},\rho_{12},\rho_{13})]=\lambda_{1}(0,0,0)=1.094$ remained above the condition for sustainability (figure 5*a*(i),*b*(i)). Even total snail elimination (*ρ*_*i*_=0) at 11, 12 and 13 did not clear the infection at those sites due to connectivity, let alone the rest of the region. In comparison with the previous strategy, a 90 per cent reduction of local BRN at sites #4, 7 and 9 that served as important transport nodes (they had close proximity to other sites) resulted in reducing *λ*_1_ below sustainability, in other words, region-wide elimination! (figure 6*c*,*d*).

## 4. Discussion

Using a mathematical model of schistosomiasis transmission for a distributed set of heterogeneous villages, we showed that the transport of parasites via social and environmental pathways has consequences for parasite control, spread and persistence. First, we found that the condition for sustainable transmission for a connected set of villages was given by the largest eigenvalue of the BRM (*λ*_1_(*R*)>1). Using a simple 2-village example, we showed that disease transmission could be sustained regionally even when individual villages did not locally support transmission. By exploring the interplay between different levels of hydrologic and social transport, we found that optimum transmission did not necessarily coincide with the most diffusive transports. Finally, we showed a rather counterintuitive finding related to disease control: in some cases, targeting of villages with high infection, without regard to village interconnections, may not lead to effective control. Sometimes even a simple understanding of inter-village connectivities may lead one to choose a more effective control strategy.

Schistosomiasis transmission provides just one example of disease persistence and emergence within interconnected populations. Migrant populations and changing interconnectivity due to increased globalization and development are important factors for many other diseases. Research on connected models for other infectious diseases has uncovered important findings. For example, similar interconnected epidemiologic models for influenza have suggested that new pandemics given the levels of modern day travel would differ significantly from historical pandemics, both in spatial-temporal distribution and in providing very little time to plan and intervene (Grais *et al*. 2004). Indeed, recent outbreaks of SARS and avian influenza provide empirical evidence of the association between modern day human connectivity and rapid disease spread, along with natural animal and plant migration and dispersal patterns (Brown & Hovmoller 2002; Olsen *et al*. 2006).

It is interesting to note that our BRM extends some earlier results on endemic equilibria in distributed environments for other patterns of connectivity (Barbour 1978; Gurarie & King 2005). More broadly, within the field of ecology, the largest eigenvalue of a suitable ‘connectivity matrix’ has been shown to play an important role in defining extinction versus colonization in metapopulations that live in fragmented environments (Hanski 2001). Despite very different formulations of connectivity (including consideration of more than one type of connectivity), the importance of the largest eigenvalue of the connectivity matrix is pervasive in these studies.

Recent modelling studies using empirical data on population mobility (Sattenspiel & Herring 2003) have shown some surprising effects of reduced connectivity (e.g. via quarantines) on the spread of disease. For instance, research on influenza transmission has found that introducing quarantine too early may not lead to the greatest reduction in disease transmission (Sattenspiel & Herring 2003). Our analyses show that reliance on traditional indicators, such as local BRN and/or prevalences, without regard to connectivity can lead to inefficient control strategies and resurgence of infection thought to be ‘under control’. Moreover, we find rather surprisingly for schistosomiasis transmission that increased social connectivity can sometimes reduce region-wide disease transmission, possibly by distributing contacts and contamination away from local hotspots for transmission. Such results point to the need for improved assessments of real-world transport and social interaction networks/processes, and further exploration of analytical methods to develop more efficient control practices.

As with all models, they are limited by the numerous assumptions upon which they are built. While we have tried to be clear in our assumptions of how hydrologic and social connectivity impact schistosomiasis transmission, there are probably many other possible implementations of the connected model. For instance, in some regions of China, bovine populations may serve as a reservoir definitive host for the parasite. Their movement from cattle grazing and trade constitutes an additional pathway through which the parasite may be transported. Similarly, the model may be made more complex through the explicit modelling of hydrological events that have occurred in the past or may be hypothesized for the future, such as periods of rainfall-runoff or drought.

Although our approach to this study is largely theoretical, our findings have practical implications, one of which has to do with the efficacy of control in connected environments. Given a distributed system, as in our figure 2, and transmission and contact parameters, one might ask to identify the ‘hotspots of transmission’ and the needed level(s) of control to achieve sustained elimination. In the same fashion, one could introduce a cost function (for different choices of ‘hotspots’ and control levels) and ask for an optimal control strategy. There is no simple mathematical formula or procedure (such as the classical BRN) to get an answer. But our numeric code allows an efficient evaluation of range of model parameters and control strategies to explore outcomes and produce optimal solutions, similar to analysis of Gurarie & King (2005) for a different type of connectivity. In that sense mathematical methods coupled with numeric procedures and modern computer tools can vastly expand the scope of systems amenable to analysis, prediction and control, beyond the classical homogeneous case and BRN.

Currently, we are limited in our ability to adequately parametrize such connected models. Most likely, connectivity parameters are site specific. As described earlier, we have made a preliminary attempt using very limited data from our study area in China to estimate reasonable values for the hydrologic and social connectivity exponents. On-going field studies from our group, as well as those of others on population mobility and migrant schistosomiasis infections, provide some hope that such work may lead to calibrated versions of these connected models. Until then, our findings herein reinforce the recommendations of others who have studied parasite transport and migrant schistosomiasis, and suggest attention be paid not only towards local variations in disease intensity, but also regional control strategies, and targeting of populations that may be particularly mobile.

## Figures and Tables

**Figure 1:**
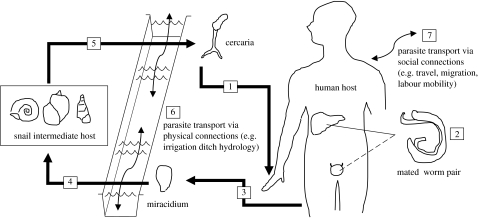
Schistosomiasis transmission. (1) Humans can become infected by the free-swimming cercarial form of the parasite from skin contact with contaminated surface water. (2) Once infected, adult schistosome worms will develop in the blood vessels of the liver or bladder depending on the species of schistosome. Male and female worms will mate and produce eggs, which are released in either the faeces or urine. (3) Inadequate sanitation results in parasitic eggs being deposited into an aquatic environment (e.g. ponds, irrigation ditches), where they hatch into a free-swimming miracidial form of the parasite. (4) Miracidia infect the snail intermediate host, which lives in these aquatic environments. (5) After a prepatency period, infected snails will begin to release cercariae, which infect humans to complete the parasite's life cycle. (6) Both cercarial and miracidial forms of the parasite may be transported between communities via hydrologic connections such as irrigation ditches. (7) Hosts may also transport the parasite between communities via travel, migration and mobile labour (infection and/or contamination within and outside of the host's own village).

**Figure 2:**
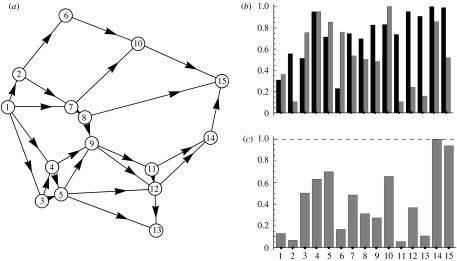
(*a*) Model environment of 15 hydrologically connected villages. Relative human and snail populations at each site are shown in (*b*) (black bars, human; grey bars, snail), along with (*c*) the resulting local basic reproduction number (local BRN)—all but site #14 are below sustainable level 1 (dashed line). The sites are numbered in the ‘down-stream’ (partial) order.

**Figure 3:**
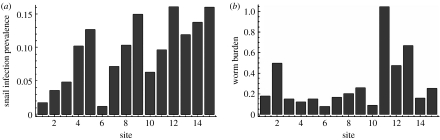
Distribution of (*a*) equilibrium snail infection prevalences (as a proportion) and (*b*) worm burdens by village. Sustained transmission occurs in all 15 villages at moderate hydrologic transport (*β*=5), wide social contact dispersion (*α*=5) and high throughput rate *r*=1, despite only site #14 in figure 1 with local BRN=1.

**Figure 4:**
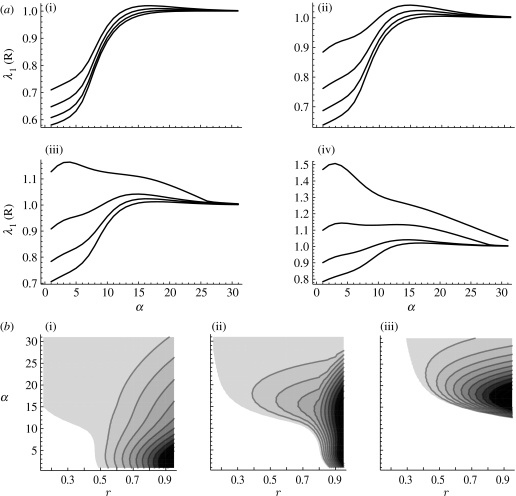
The effect of social and hydrological connectivities on persistence of endemic infection. (*a*) Eigenvalue *λ*_1_(*R*) over the range of social contact dispersion 1<*α*<30 for four hydrologic transport *β*=4; 5; 6; 7 (top-to-bottom curves). (i–iv) follow changing hydrologic throughput from low throughput *r*=0.4 (i) to high throughput *r*=1 (iv). (*b*(i)–(iii)) shows the isocontours of the eigenvalue *λ*_1_(*R*) in the (*r*,*α*) parameter space for three values of *β*=2 (i) to *β*=6 (iii) (shaded isocontours represent *λ*_1_(*R*)>1 with darker shades of grey indicating higher values *λ*_1_(*R*)). In (*a*,*b*), we observe how different choices of hydrologic parameters *r*, *β* create distinct ranges of social contact dispersion *α* for persistent infection (*λ*_1_(*R*)>1).

**Figure 5:**
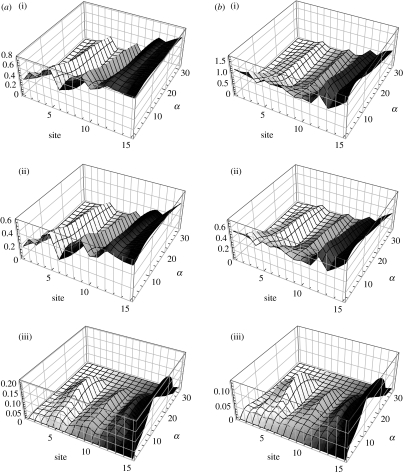
(*a*(i)–(iii)) Equilibrium snail infection prevalences and (*b*(i)–(iii)) rescaled worm burdens over 15 village sites for varying levels of social contact dispersion 1<*α*<30, at high throughput *r*=0.9. (*a*(i)–*b*(i)) The snail infection prevalences and worm burdens for *β*=2 (fast hydrologic transport), (*a*(ii)–*b*(ii)) *β*=3 (medium hydrologic transport) and (*a*(iii)–*b*(iii)) *β*=5 (slow hydrologic transport).

**Figure 6:**
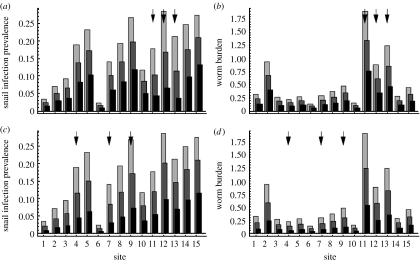
Local molluscicide control. (*a*,*c*) Snail infection prevalence and (*b*,*d*) worm burden. (*a*,*b*) Focal control at sites #11, 12 and 13: at 100% snail density (light grey), 50% reduction (dark grey), 0% complete removal (black). (*c*,*d*) Similar control at sites #4, 7 and 9 with 100% snail density (light grey), and reductions of 30% (dark grey), 60% (black) and 90% (invisible—elimination).

**Table 1:** Definition of basic model variables and parameters.

variables	*w*	mean worm burden (i.e. the mean number of schistosomes per person)
	*y*	prevalence (as a proportion of *N*) of patent infectious snails
	*c*	cercarial density (per unit habitat)
	*m*	miracidial density (per unit habitat)
	*H*	number of humans
parameters	*N*	number of intermediate snail hosts
	*a*	probability of cercarial infection and establishment in humans
	*b*	rate of miracidial infection and establishment in snails
	*θ*, *θ*′	contact rate for exposure and contamination
	*π*_C_	rate of cercarial output per infected snail host
	*π*_M_	rate of miracidial output per mated worm pair (in human host)
	*μ*_C_	*per capita* mortality rate for cercariae
	*μ*_M_	*per capita* mortality rate for miracidia
	*γ*	*per capita* mortality rate for schistosome worms (in human)
	*ν*	*per capita* mortality rate for snails
	*A*	snail–human transmission coefficient
	*B*	human–snail transmission coefficient
	*R*_0_	basic reproduction number (BRN) for schistosomes

**Table 2:** Connectivity (hydrology and social contact) parameters.

$\left\{\theta_{i};\theta_{i}^{\prime }\right\}$	site-specific contact rates (for exposure and contamination)
$\omega_{ij}\propto {\text{e}}^{-\alpha d_{ij}}$	contact distribution fractions (from *j* to *i* site)
*α*	exponent for social contact dispersal (increased *α* means ‘lesser connectivity’)
$\Omega =[\omega_{ij}]$	spatially explicit social contact matrix (with entries *ω*_*ij*_)
{*V*_*i*_}	fixed reservoir volumes
*r*_*ij*_	fixed hydrologic throughput rate from *j* to *i* (per unit volume)
$r_{i}=\sum_{i≺j}r_{ji}$	total outflow (discharge) rate at site *i* (per unit volume). Symbol i≺j indicates a ‘down-stream’ relationship (i.e. village *j* downstream of village *i*).
*ϵ*	relative larval loss rate fraction due to discharge (outflow)
$s_{ij}^{\text{C}}={\text{e}}^{-\beta_{\text{C}}d_{ij}}$	transport survival rates for cercaria in the ‘*j*–*i*’ channel
$s_{ij}^{\text{M}}={\text{e}}^{-\beta_{\text{M}}d_{ij}}$	transport survival rates for miracidia
*β*_C_; *β*_M_	exponents for hydrologic transport survival for cercaria and miracidia (increased *β* implies diminished survival, due to ‘slower hydrology’)
*T*_C_	spatially explicit transport matrix for cercaria that incorporates hydrological connectivity, survival and mortality
*T*_M_	spatially explicit transport matrix for miracidia that incorporates hydrological connectivity, survival and mortality
$\hat{A}$	diagonal matrix of snail–human local transmission coefficients
$\hat{B}$	diagonal matrix of human–snail local transmission coefficients
*R*_HS_	human–snail connectivity matrix
*R*_SH_	snail–human connectivity matrix
$\hat{\rho }$	diagonal matrix of the local BRN (*R*_0_) for each village
*R*	basic reproduction matrix (BRM) for the connected system
*λ*_1_(*R*)	largest eigenvalue of the basic reproduction matrix *R*

**Table 3:** The basic (relative) parameters of the model environment (figure 2). (Contact rates for exposure and contamination are assumed to be the same.)

site no	1	2	3	4	5	6	7	8	9	10	11	12	13	14	15
*H*_*i*_	0.31	0.56	0.51	0.95	0.71	0.23	0.75	0.7	0.83	0.83	0.74	0.95	0.91	1.0	0.99
*N*_*i*_	0.36	0.11	0.76	0.95	0.85	0.76	0.54	0.5	0.48	1.0	0.1	0.24	0.16	0.86	0.52
*θ*, *θ*′	0.8	0.79	0.84	0.62	0.79	0.73	0.81	0.7	0.61	0.66	0.63	0.93	0.64	0.8	1.0

**Table tbl4:** 

site #	downstream connections
1	2,3,4,7
2	6,7
3	4,5
etc.	
